# Association of indoor microbial aerosols with respiratory symptoms among under-five children: a systematic review and meta-analysis

**DOI:** 10.1186/s12940-021-00759-2

**Published:** 2021-07-01

**Authors:** Adekunle Gregory Fakunle, Nkosana Jafta, Rajen N. Naidoo, Lidwien A. M. Smit

**Affiliations:** 1 Discipline of Occupational and Environmental Health, School of Nursing and Public Health, University of KwaZulu-Natal, Durban, South Africa; 2 Department of Medicine, University of Ibadan, Ibadan, 200284 Nigeria; 3 Institute for Risk Assessment Sciences, Environmental Epidemiology Division (IRAS-EEPI), Utrecht University, Utrecht, The Netherlands

**Keywords:** Indoor microbiome, Respiratory symptoms, Asthma, Under-five children, Meta-analysis

## Abstract

**Background:**

Despite the recognition of the importance of indoor microbial exposures on children’s health, the role of different microbial agents in development and aggravation of respiratory symptoms and diseases is only poorly understood. This study aimed to assess whether exposure to microbial aerosols within the indoor environment are associated with respiratory symptoms among children under-5 years of age.

**Methods:**

A systematic literature search was conducted on PubMed, Web of Science, GreenFILE, ScienceDirect, EMBASE and Cochrane library through February 2020. Studies that investigated the exposure–response relationship between components of the indoor microbial communities and respiratory symptoms among under-five children were eligible for inclusion. A random-effect meta-analysis was applied to estimate pooled relative risk (RR) and 95% confidence interval (CI) for study specific high versus low microbial exposures. The potential effect of individual studies on the overall estimate was evaluated using leave-one-out analysis, while heterogeneity was evaluated by *I*^2^ statistics using RevMan 5.3.

**Results:**

Fifteen studies were eligible for inclusion in a meta-analysis. The pooled risk estimate suggested that increased microbial exposure was associated with an increased risk of respiratory symptoms [pooled relative risk (RR): 1.24 (1.09, 1.41), *P* = 0.001]. The association was strongest with exposure to a combination of *Aspergillus*, *Penicillium, Cladosporium and Alternaria* species [pooled RR: 1.73 (1.30, 2.31), *P* = 0.0002]. Stratified analysis revealed an increased risk of wheeze [pooled RR: 1.20 (1.05, 1.37), *P* = 0.007 and allergic rhinitis [RR: 1.18 (0.94, 1.98), *P* = 0.16] from any microbial exposure.

**Conclusions:**

Microbial exposures are, in general, associated with risk of respiratory symptoms. Future studies are needed to study the indoor microbiome more comprehensively, and to investigate the mechanism of these associations.

**Supplementary Information:**

The online version contains supplementary material available at 10.1186/s12940-021-00759-2.

## Background

The microbial community within indoor environments such as dwellings where humans, especially under-five children, spend more than 90% of their time, consists of a wide range of microorganisms including bacteria, fungi, and viruses [1–4]. The microbial load and composition within the indoor environment is determined and influenced by the presence, identity and activities of human occupants [5–7]. Non-human occupants, such as dogs [8, 9] and household insects [10], can also influence the microbial profile of the indoor environment. In addition, indoor microbial communities can be influenced by differences in ventilation, building design, the environmental characteristics found within buildings [9, 11, 12] or prior water damage [13]. The interest in the indoor microbiome has increased over the last few decades [1]. This is largely because of the wider recognition that exposures to microbes in the residential indoor environment are associated with a vast number of adverse health outcomes with major public health importance, including infectious diseases, acute toxic effects, allergies and cancer [1].

Most epidemiological studies have been heterogeneous in assessing different respiratory health effects associated with exposure to specific microbial components, especially endotoxins, and culturable molds [14–17] while very few have investigated indoor microbial communities [18, 19]. These studies have shown inconsistent findings, suggesting protective, detrimental, and no health effects in relation to asthma and allergy [14]. Greater diversity of fungal and bacterial agents has been shown to reduce the risk of asthma and wheeze in children [17, 20, 21] while others found a positive association between elevated levels of total viable mold and risk of rhinitis with persistent cough [22–24].

Respiratory health effects have been the subject of recent research among preschool children [25, 26] and children under the age of 5 years [27]. Children under the age of 5 years are more at risk of respiratory outcomes from exposure to indoor microbial agents due to the fact that they spend a considerable proportion of time in the home environment during a period of intense growth and development of the immunologic and respiratory systems [28, 29]. Despite the recognition of the importance of exposure to the indoor microbiome on children’s health, the precise role of different microbial agents in the development and aggravation of symptoms and diseases is only poorly understood. It is therefore not clear which specific component(s) primarily contribute to the presumed respiratory health effects. To the best of our knowledge, there has been no systematic review or meta-analysis exploring the role of the indoor microbial exposure on respiratory health outcomes among children under 5. Therefore, this systematic review and meta-analysis aims to summarize evidence of associations between different indoor microbial agents and their combined role in the incidence of respiratory allergies and asthma, thereby providing opportunities to improve future respiratory health interventions among under-five children.

## Methods and design

### Literature search

The protocol for this meta-analysis was reported using MOOSE guideline [30] and registered in the International Prospective Register of Systematic Reviews (Reg ID: CRD42020178514). Two reviewers independently explored PUBMED, WEB OF SCIENCE, GREEN FILE (EBSCO), ScienceDirect, EMBASE and Cochrane databases independent of date through February 2020 to identify appropriate previously published studies using the following search terms; “home” OR “house” OR “dwelling” OR “residence” OR “residential” OR “indoor” OR “domicile” OR “living unit” OR “property” OR “build” OR “built environment” OR “domestic environment” OR “bedroom” OR “living room” OR “wall” OR “floor” OR “ceiling” OR “construction material” **AND** “damp” OR “fungi” OR “mold” OR “mould” OR “fungal” OR “fungus” OR “bacteria” OR “virus” OR “microbial” OR “microbiome” OR “microbial diversity” OR “microbial load” OR “microbial burden” OR “microbiota” OR “biodiversity” **AND** “respiratory symptoms” OR “allergy” OR “hay fever” OR “cough” OR “fever” OR “difficulty breathing” OR “wheeze” OR “allergic rhinitis” OR “sinusitis” OR “asthma”. Title and abstract of each article was evaluated independently and differences on which publication(s) to include were clarified by recourse to a third reviewer. A snowball search was also carried out by screening reference lists of publications and reviews.

### Inclusion criteria and study selection

Studies eligible for inclusion in the meta-analysis were epidemiological reports in humans with exposure–response relationship between indoor microbial aerosols and respiratory symptoms. Inclusion criteria were: (I) qualitative or quantitative assessment of indoor microbial aerosols. Studies that reported exposure to specific microbial communities (bacteria, fungi, viruses, and/or microbial by-products) or presence/absence of visible molds were included; (II) studies conducted among children aged ≤ 5 years; (III) respiratory symptoms (including wheeze/allergic rhinitis) and/or asthma adequately defined and described; (IV) full text articles in English originally published in peer reviewed journals. Literature reviews, abstracts, letters to the editor, case reports, and non-human studies were excluded.

Assessment of articles was performed in EndNote databases. All duplicates were removed and studies were selected based on title or abstract for full text-screening. For studies that were excluded, the reasons for exclusion were listed.

### Data extraction

From the studies that met the inclusion criteria, the following information was extracted: (1) name and initials of the first author, (2) year of publication, (3) country, (4) type of sample, (5) sampling equipment, (6) microbial agent(s), (7) analytical method, (8) level and measure of exposure, (9) respiratory outcome definition, (10) number of cases, (11) sample size and (12) effect estimate. All incongruities from the data extracted were resolved by a third author.

### Quality assessment of included studies

Two team members (AGF and NJ) assessed the methodological quality and risk of bias of the included studies using the Cochrane Collaboration guidelines [31] and the Newcastle–Ottawa scales [32]. The quality of the studies was graded by rating nine items representing the study selection procedure, comparability, and outcome/exposure definition. Each item was scored as ‘yes’ (if present) and ‘no’ (if absent) in the included studies and the overall scores were presented as percentages. Studies with median a score ≥ 80% (median in our study) were arbitrarily considered to have a low risk of bias while those with a score < 80% were considered to have a high risk of bias.

### Statistical analyses

All statistical analysis was carried out using Review Manager 5.3. We applied the inverse of variance method for weighting and computed the summary effect estimates by first log-transforming all relative risk (RR) and 95% confidence interval (CI) for high vs low category (as reference) of microbial exposure. The standard error (SE) was estimated based on the formula:

1 $${\text{SE}} = \left[ {{\text{log }}\left( {{\text{upper limit of the 95}}\% {\text{ CI}}} \right){-}{\text{log }}\left( {{\text{lower limit of the 95}}\% {\text{ CI}}} \right)/{\text{3}}.{\text{92}}} \right]$$

The extent of variability across studies and heterogeneity of the summary effect estimates were evaluated using *I*^*2*^ test statistics. Where *I*^*2*^ statistics ≥ 50%, a random effect model was employed, otherwise, a fixed effect model [30, 33]. The area of the black square in forest plots implies the weighted contribution by each study. Sensitivity analyses of the results and publication bias was evaluated using leave-one-out and funnel plot techniques respectively. *P* < 0.05 (two-tailed) was considered statistically significant [34].

## Results

**Fig. 1:**
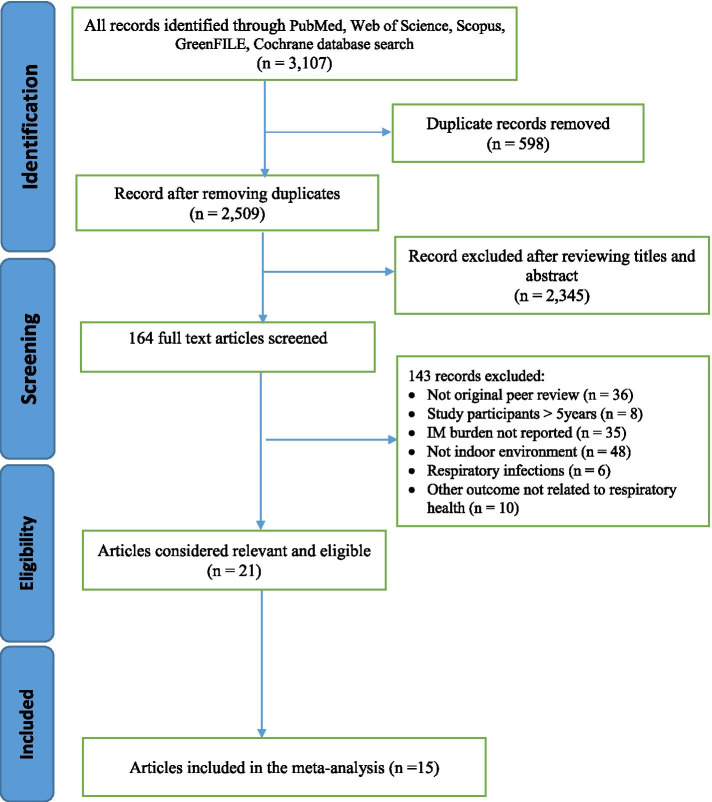
PRISMA Flow chart of the literature search for studies investigating Exposure to IM and respiratory symptoms among U-5C. IM = Indoor Microbiome; U-5C = Under-five Children

The PRISMA flowchart describing the process used to identify the studies eligible for our meta-analyses yielded 3,107 records (Fig. 1). In addition, duplicates (*n* = 598) and other records (*n* = 2,345) after screening titles and abstracts were excluded, resulting in 164 articles for full text assessment. In total, fifteen articles that fulfilled all inclusion criteria were included in the meta-analyses [23, 35–48].

Detailed characteristics of the included studies are presented in Tables 1 and 2. All fifteen studies had a cohort design with follow-up period between 1 [35, 37–39, 41, 42, 48] and 4 [40, 47] years. Eleven studies were carried out in the United States and four in Europe all between 2000 and 2019. All studies included examined indoor microbial exposure among children under the age of 5 years. The methods of assessment of exposure to indoor microbial aerosols used among studies included air sampling [35, 39], dust sampling [37, 38, 40–44, 46–48] and home inspection using a standard observational checklist [45]. In addition, two studies [23, 36] applied a combination of air and dust sampling techniques. The indoor air samples were collected using Burkard portable air sampler [35] and Hirst-type sampler [39] respectively. The dust samples were collected using high volume vacuum cleaners such as Eureka Mighty-Mite vacuum cleaner, Filter Queen Majestic vacuum cleaner and Rowenta Dymbo vacuum cleaner. Microbial agents assessed were total bacteria [36], total fungi [23, 36, 39], specific fungal genera such as *Aspergillus spp*. [23, 36, 39], *Penicillium spp.* [23, 35, 36, 39], *Cladosporium spp.* [23, 35, 36, 39]*, Alternaria spp*. [23, 36], yeast [23, 36, 49], visible molds [23, 45] and endotoxins [37, 38, 40–44, 46–48]. In terms of the analytical methods employed, the bacterial and fungal agents were assessed using conventional plate count methods [23, 35, 36] while endotoxins were analysed using the kinetic chromogenic Limulus amebocyte lysate (LAL) test [41–44, 46–48]. Respiratory outcomes in the included studies were allergic rhinitis [23, 42, 43, 45], wheeze [35–44, 46–48] and asthma [44, 47]. These outcomes were assessed by doctors’ diagnosis using clinical examination [23, 39, 47] and parental/caregiver’s recall [35–38, 40–46, 48]. After a detailed quality assessment of the included studies based on the Newcastle–Ottawa scale for assessing the quality of non-randomized studies, most of the studies (*n* = 8) had a score < 80% (median in our study) and were categorized as high risk of bias. Others (*n* = 7) were considered to have a low risk of bias (score ≥ 80%) (Table S1).

**Table 1:** Participants characteristics of eligible studies included in the meta-analysis

**Author**	**Year**	**Country**	Microbial assessment/definition of measure					**Assessment of Respiratory health Outcome**			**Effect estimate**
			**Type of sample**	**Sampling equipment**	**Agent**	**Analytical method**	**Measure of Exposure**	**Definition**	**N Cases**	**N total (baseline)**	
**Gent et al.,**	2002	USA	Indoor Air	Burkard portable air sampler	Fungi	Plate count;	Undetectable® **Vs** ≥ 1000 cfu/m^3^	Reported wheeze and persistent cough measured as number of days of symptoms (0, < 30 and ≥ 30 days)	119	880	RPW: 2.15 (1.34 – 3.46)^a^ PC: 2.06 (1.31 – 3.24)^a^
**Rosenbaum et al.,**	2010	USA	Indoor Air; Dust sample	Single-stage Andersen air sampler; High volume vacuum cleaner	Bacteria; Fungi; Endotoxin	Plate count; KLARE	Not detectable® **Vs** > 75^th^ percentile	Primary care provider documented wheeze; Wheeze heard on physical examination by a clinician	39	103	6.18 (1.34—28.46)^a^
**Horick et al.,**	2006	USA	Dust sample	High volume vacuum cleaner	Endotoxin	KLARE	TEC < 100 EU/mg® **Vs** ≥ 100EU/mg	Primary care provider reported “any wheeze” ≥ 1 episode	42	360	5.56 (1.19 – 26.03)^a^
**Park et al.,**	2001	USA	Dust sample	Eureka Mighty-Mite vacuum cleaner	Endotoxin	KLARE	TEC < 100 EU/mg® **Vs** ≥ 100EU/mg	Primary care provider reported “any wheeze” ≥ 1 episode or repeated episode of wheeze	211	499	AW: 1.33 (0.99–1.79)^a^ RW: 1.55 (1.00 – 2.42)^a^
**Harley et al.,**	2009	USA	Indoor Air	Hirst-type sampler	Fungi spores	Spore count	Not detectable® **Vs** ≥ 1000 cfu/m^3^	A child was considered to have early wheezing if medical records indicated a clinician’s diagnosis of asthma at any time between birth and 24 months of age	35	514	1.2 (0.7 – 2.7)^a^
**Litonjua et al.,**	2002	USA	Dust sample	High volume vacuum cleaner	Endotoxin	KLARE	TEC < 81.3 EU/mg® **Vs** ≥ 81.3EU/mg	Wheezing in the past 12 month or repeated wheezing twice in the 4 years of follow-up	57	226	W: 1.52 (1.07—2.14)^a^ RW: 2.57 (1.00 – 6.62)^a^
**Campo et al.,**	2006	USA	Dust sample	Filter Queen Majestic vacuum cleaner	Endotoxin	Kinetic Chromogenic -LAL	TEC < 100 EU/mg® **Vs** ≥ 100EU/mg	Recurrent wheeze; ≥ 2 wheezing episodes in the past 12 months or any wheezing: ≥ 1 wheezing episode in the past 12 months	137	532	RCW: 0.4 (0.1–0.9)^a^ AW: 0.3 (0.1–0.8)^a^
**Gillespie et al.*,***	2006	New Zealand	Dust sample	High volume vacuum cleaner	Endotoxin	Kinetic Chromogenic -LAL	TEC < 100 EU/mg® **Vs** ≥ 100EU/mg	Reported wheeze for at least 6 months. Rhinitis defined as blocked or runny nose when the child do not have a cold or flu	342	881	1.54 (1.03 – 2.30)^a^
**Perzanowski et al.*,***	2006	USA	Dust sample	Eureka Mighty-Mite vacuum cleaner	Endotoxin	Kinetic Chromogenic -LAL	TEC < 100 EU/mg® **Vs** ≥ 100EU/mg	Wheezing defined as presence of runny nose, sneezing, itchy eyes without cold at age 12, 24 and 36 months	163	301	1.04 (0.71 – 1.5)^a^
**Karvonen et al.*,***	2012	EU	Dust sample	High volume vacuum cleaner	Endotoxin	Kinetic Chromogenic -LAL	TEC < 100 EU/mg® **Vs** ≥ 100EU/mg	Primary care provider reported “any wheeze” ≥ 1 episode	984	1133	0.71 (0.51 – 0.99)^a^
**Stark et al.,**	2005	USA	Indoor Air Dust sample	Burkard portable air sampler; Eureka Mighty Mite canister vacuum cleaner	Fungi	Plate count	Low® **Vs** High TEC < 100 EU/mg® **Vs** ≥ 100EU/mg	Doctor diagnosed allergic rhinitis or hay fever	52	405	3.13 (1.51 – 6.47)^a^
**Biagini et al.,**	2006	USA	Home inspection	Walkthrough Checklist	Mold	Observation	Low® **Vs** High	Rhinitis defined as parents’ report of sneezing or a runny or blocked nose not associated with a cold or chest infection’ in the past 30 days	242	495	1.7 (0.7 – 3.8)^a^
**Bolte et al.,**	2003	Germany	Dust sample	High volume vacuum cleaner	Endotoxin	Kinetic Chromogenic -LAL	Highest quartile **Vs** Lowest quartile®	Repeated wheeze defined as having had at least 2 episode of wheezing	378	1,942	1.77 (1.14 – 2.73)^a^
**Douwes et al.,**	2006	The Netherlands	Dust sample	Rowenta Dymbo vacuum cleaner	Endotoxin	Kinetic Chromogenic -LAL	Highest quartile **Vs** Medium quartile®	Doctor-diagnosed asthma was defined as a reported diagnosis confirmed by a doctor at any time in the past 4 years	547	696	0.40 (0.21 – 0.77)^a^
**Phipatanakul et al.,**	2005	USA	Dust sample	High volume vacuum cleaner	Endotoxin	Kinetic Chromogenic -LAL	4^th^ quartile **Vs** 1^st^ quartile®	Any report of wheeze (any wheeze) in the first year of life	197	498	2.39 (1.22 – 4.68)^a^

*YOF* Year of Follow-up, *EU* Europe, *NA* Not applicable, *W* Wheeze in the past 12 months, *KLARE* Kinetic Limulus assay with the resistant-parallel-line estimation, *LAL* Limulus amebocyte lysate, *AW* Any wheeze, *RW* Repeated wheeze, *RPW* Reported wheeze, *RCW* Recurrent wheeze, ® Reference value, *PC* Persistent cough, *SPT* Skin Prick Test

^a^Multivariable adjusted effect estimate for LRTI risk

**Table 2:** Summary effect estimate for the relationship between any IM exposure (highest estimates in the studies) and Respiratory symptoms (*n* = 15), and stratified analysis according to study characteristics

Stratification	**Study Characteristics (Number of studies)**	***I***^***2***^*** (%)***	**Summary Effect Estimate for pooled adjusted data [95% CI]**	***P*-value**
Population	All studies (15) [12, 18, 25–37]	78	1.24 [1.09, 1.41]	0.001
Study size^a^	Large (6) [25, 30, 32, 34–36]	82	1.14 [1.02, 1.38]	0.04
	Small (9) [12, 18, 26–29, 31, 33, 37]	67	1.33 [1.11, 1.59]	0.002
Geographical Location	United States (11) [12, 18, 25–31, 33, 37]	63	1.35 [1.15, 1.57]	0.0001
	Europe (4) [32, 34–36]	81	1.06 [0.87, 1.28]	0.56
Year of Publication	2010 – 2019 (2) [12, 34]	84	1.34 [0.58, 3.09]	0.49
	2000 – 2009 (13) [18, 25–33, 35–37]	60	1.25 [1.12, 1.41]	0.0002
Method of IM Assessment	Air sampling (3) [12, 25, 28]	2	1.48 [1.25, 1.75]	0.00001
	Dust sampling (11) [18, 26, 27, 29, 31–37]	76	1.12 [0.98, 1.29]	0.11
	Home Inspection (1) [30]	NA	1.66 [0.85, 3.21]	0.14
Method of diagnosis	Doctor diagnosed (2) [18, 28]	NA	1.60 [1.28, 2.01]	< 0.0001
	Self-reported (13) [12, 25–27, 29–37]	76	1.19 [1.04, 1.35]	0.009
Study Quality	Low risk of bias (7) [12, 18, 25, 27, 28, 31, 32]	59	1.31 [1.12, 1.55]	0.001
	High risk of bias (8) [26, 29, 30, 33–37]	75	1.17 [0.99, 1.37]	0.06

*NA* Not applicable

^a^A large study was defined as a cohort study with a sample size of greater than 600

**Fig. 2:**
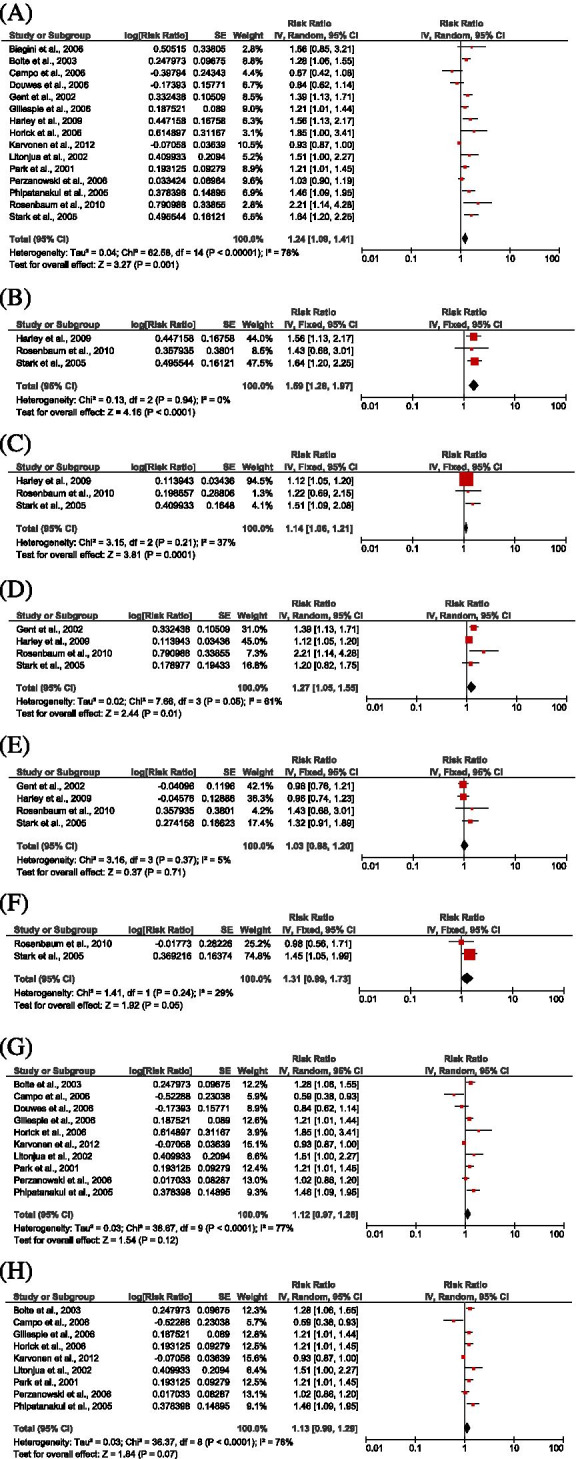
Forest plot for the relationship between any IM exposure and Respiratory symptoms among U-5C with pooled effect estimates (**A**); between TFC and Respiratory symptoms (**B**); between *Aspergillus* species and Respiratory symptoms (**C**); between *Penicillium* species and Respiratory symptoms (**D**); between *Cladosporium* species and Respiratory symptoms (**E**); between *Alternaria* species and Respiratory symptoms (**F**); between Endotoxin and Respiratory symptoms (**G**); between Endotoxin and Wheeze (**H**)

Tables 2 and 3 provided study characteristics and summary effect estimates of all 15 studies [23, 35–48] addressing the association between different indoor microbial exposures and respiratory symptoms among children under 5. The pooled risk estimate from the random effect model showed a significant association between microbial exposure and respiratory symptoms; RR: 1.24 (1.09, 1.41), *P* = 0.001, *I*^2^ = 78% (Fig. 2A). The risk estimate was observed to vary geographically: RR: 1.35 (1.15, 1.57), *P* = 0.0001; *I*^2^ = 63% (United States) and RR: 1.06 (0.87, 1.28, *P* = 0.56; *I*^2^ = 81% (Europe). Further stratification showed that the risk estimates were RR: 1.48 (1.25, 1.75), *P* < 0.00001 for studies that used air sampling for exposure assessment and RR: 1.12 (0.98, 1.29), *P* = 0.11 for studies based on dust sampling techniques. Also, indoor microbial exposure was found to be associated with an increased risk of wheeze independent of the risk of bias of studies included in the meta-analysis.

**Table 3:** Effect estimates (EEs) of studies for the association between IM and respiratory symptoms among U-5C (the highest EEs reported for any IM exposure)

**Author, year/Country**	**Type of Exposure and EEs** **Adjusted EE (95% CI)**							
	**Any IM exposure**	**TFC**	***Aspergillus spp.***	***Penicillium spp.***	***Cladosporium spp.***	***Alternaria spp.***	**Visible molds**	**Endotoxin**
Gent et al., 2002/USA [35]	2.15 (1.34, 3.46)	-	-	2.15 (1.34, 3.46)	0.91 (0.53, 1.56)	-		-
Rosenbaum et al., 2010/USA [36]	6.18 (1.34, 28.46)	3.64 (0.67, 19.65)	1.58 (0.43, 5.79)	6.18 (1.34, 28.46)	2.28 (0.41, 12.67)	0.96 (0.27, 3.45)		-
Horick et al., 2006/USA [37]	4.12 (1.03, 16.83)	-	-	-	-	-		4.12 (1.03, 16.83)
Park et al., 2001/USA [38]	1.56 (1.03, 2.38)	-	-	-	-	-		1.56 (1.03, 2.38)
Harley et al., 2009/USA [39]	2.80 (1.30, 5.90)	1.20 (0.70, 2.00)	1.3 (1.10, 1.50)	1.3 (1.10, 1.50)	0.90 (0.50, 1.60)	-		-
Litonjua et al., 2002/USA [40]	2.57 (1.00, 6.62)	-	-	-	-	-		2.57 (1.00, 6.62)
Campo et al., 2006/USA [41]	0.40 (0.10, 0.90)	-	-	-	-	-		0.40 (0.10, 0.90)
Gillespie et al. 2006/Europe [42]	1.54 (1.03, 2.30)	-	-	-	-	-		1.54 (1.03, 2.30)
Perzanowski et al. 2006/USA [43]	1.04 (0.71, 1.50)	-	-	-	-	-		1.04 (0.71, 1.50)
Karvonen et al. 2012/Europe [44]	0.85 (0.72, 1.00)	-	-	-	-	-		0.85 (0.72, 1.00)
Bolte et al., 2003/Europe [46]	1.77 (1.14, 2.73)	-	-	-	-	-		1.77 (1.14, 2.73)
Douwes et al., 2006/Europe [47]	0.40 (0.21, 0.77)	-	-	-	-	-		0.40 (0.21, 0.77)
Phipatanakul et al., 2005/USA [48]	2.39 (1.22, 4.68)	-	-	-	-	-		2.39 (1.22, 4.68)
Stark et al., 2005/USA [23]	3.13 (1.51, 6.47)	3.13 (1.51, 6.47)	2.57 (1.22, 4.40)	1.51 (0.63, 3.64)	1.88 (0.81, 4.35)	2.34 (1.12, 4.91)	1.66 (0.87, 3.17)	-
Biagini et al., 2006/USA [45]	1.70 (0.70, 3.80)	-	-	-	-	-	1.70 (0.70, 3.80)	-

*TFC* Total Fungal Count

Adjusted model in each study:

- Gent et al., adjusted for socioeconomic factors and housing characteristics

- Rosenbaum et al., adjusted for season of visit, maternal smoking during pregnancy, any smoker in the home, day care center or nonrelative care, and endotoxin

- Horick et al., adjusted for race, presence of dog in home, former (not current) dog in home, use of dehumidifier, total mass of dust sample collected (in log scale), presence of concrete floor, missingness indicator for presence of concrete floor, and presence of water damage

- Park et al., adjusted for age, race/ethnicity and socioeconomic characteristics

- Harley et al., adjusted for gas stove in home, respiratory infection in first year of life, and PM_2.5_ in first 3 months of life (residuals independent of spores)

- Litongua et al., adjusted for maternal asthma, maternal age, sex, prematurity, and area of residence

- Campo et al., adjusted for sex, daycare attendance, number of siblings, mother smokes, parental history of asthma

- Gillespie et al., adjusted for household size, number of rooms in the house, pet in home, dampness, musty smell, maternal smoking, open fireplace, type of flooring in the bedroom, and New Zealand Deprivation index

- Perzanowski et al., adjusted for sex, maternal asthma, ethnicity and tobacco smoke exposure in the home

- Karvonen et al., adjusted for study centre, farming status, gender, maternal history of allergic disease, smoking during pregnancy and number of sibling

- Bolte et al., adjusted for gender, study region, breastfeeding, elder siblings, parental education, mite and cat allergen levels, frequent respiratory infections and smoking during pregnancy

- Douwes et al., adjusted for sex, region, parental education level, exposure to indoor tobacco smoke in the past 4 years, and other children in the household at 4 years of age

- Phipatanakul et al., adjust for sex, household income, and paternal history of asthma

- Stark et al., adjusted for water damage or mold or mildew in year 1, African-American ethnicity, maternal Alternaria, IgE > 0.35 U/mL

- Biagini et al., adjusted for mother’s education, gender, cat and dog ownership, daycare attendance, breastfeeding and number of diaries returned

Three studies [23, 36, 39] provided study-specific estimates for risk of respiratory symptoms from exposure to total fungal concentration; TFC (CFU/m^3^), resulting in a risk estimate of RR: 1.59 [1.28. 1.97], *P* < 0.0001; *I*^2^ = 0% (Fig. 2B). Risk of respiratory symptoms based on exposure to specific fungal genera revealed a pooled risk estimate of RR: 1.14 [1.06, 1.21], *P* = 0.0001 for *Aspergillus* species (Fig. 2C), RR: 1.27 [1.05, 1.55], *P* = 0.01 for *Penicillium* species (Fig. 2D), RR: 1.03 [0.88, 1.20], *P* = 0.71 for *Cladosporium* species (Fig. 2E) and RR: 1.31 [0.99, 1.73], *P* = 0.05 for *Alternaria* species (Fig. 2F). The combined model based on four studies [23, 35, 36, 39] showed a significantly increased risk of respiratory symptoms when under-five children were exposed to a combination of two most reported fungal genera; *Penicillium spp.* and *Cladosporium spp*. compared to unexposed under-five children [RR: 1.51 (1.31, 1.76), *P* < 0.00001; *I*^2^ = 0%] (model 1). Three studies [23, 36, 39] revealed that exposure to a combination of three commonly reported fungal genera; *Aspergillus spp*., *Penicillium spp*. and *Cladosporium spp*. produced a stronger risk of respiratory symptoms [RR: 1.66 (1.34, 2.06), *P* < 0.00001; *I*^2^ = 0%] (model 2). The study-specific estimates based on two studies [23, 36] showed that exposure to a combination of *Aspergillus spp*., *Penicillium spp*., *Cladosporium spp.* and *Alternaria spp*. significantly increase the risk of respiratory symptoms by 73% [RR: 1.73 (1.30, 2.31), *P* = 0.0002; *I*^2^ = 0%] (model 3) (Table 4). Ten studies [37, 38, 40–44, 46–48] investigated the relationship between endotoxins and respiratory symptoms, resulting in a pooled estimate that was not statistically significant [1.12 (0.97, 1.28), *P* = 0.12, *I*^2^ = 77%] (Fig. 2G) likewise the relationship between endotoxins and wheeze among under-five children (Fig. 2H) obtained from a pooled risk estimate of nine studies.

**Table 4:** Combined effect estimate for the relationship between exposure to fungal genera and respiratory symptoms among U-5C

Model in subgroup analysis	**Number of studies**	***I***^***2***^*** (%)***	**Summary Effect Estimate for pooled adjusted data** **[95% CI]**	***P*-value**
**Model 1:** Two most reported fungal genera; *Penicillium* and *Cladosporium species*	(4)	0	1.51 [1.31, 1.76]	< 0.00001
**Model 2:** Three commonly reported fungal genera; *Aspergillus*, *Penicillium, Cladosporium*	(3)	0	1.66 [1.34, 2.06]	< 0.00001
**Model 3:** Four reported fungal genera; *Aspergillus*, *Penicillium, Cladosporium and Alternaria*	(2)	0	1.73 [1.30, 2.31]	0.0002

**Fig. 3:**
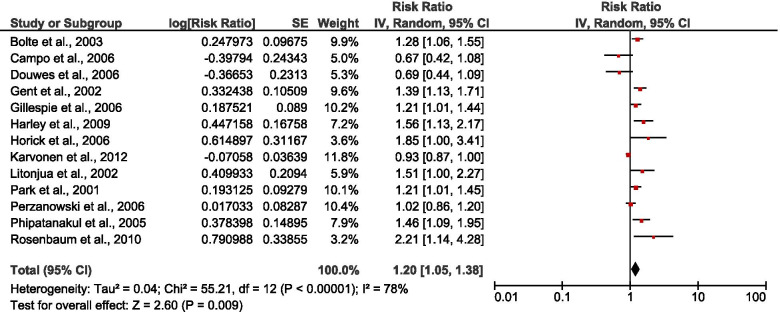
Forest plot for the relationship between any IM exposure and wheeze among U-5C with pooled effect estimates

**Fig. 4:**
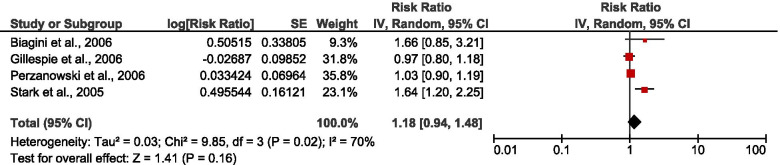
Forest plot for the relationship between any IM exposure and allergic rhinitis among U-5C with pooled effect estimates

**Fig. 5:**
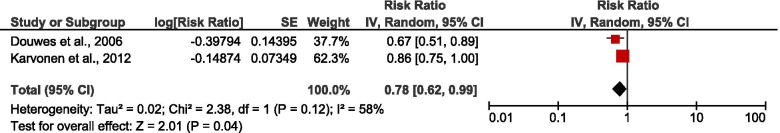
Forest plot for the relationship between endotoxin and asthma among U-5C with pooled effect estimates

Thirteen studies [35–44, 46–48] assessed the association between different microbial exposures and wheeze. The pooled risk estimate from the random effect model showed a significant association between indoor microbial exposure and wheeze [RR: 1.20 (1.05, 1.37), *P* = 0.007, *I*^2^ = 78% (Fig. 3). Only four studies [23, 42, 43, 45] investigated the association between different microbial exposure and allergic rhinitis with no significant relationship (Fig. 4). In addition, two studies [44, 47] investigated the association between any exposure and asthma among under-five children with a significant protective effect [RR: 0.78 (0.62, 0.99), *P* = 0.04], (Fig. 5).

The funnel plots showed no significant evidence of publication bias among the studies included in the meta-analyses (Supplementary Figures S1, S2 and S3). We tested the effect of excluding individual studies on the stability of the pooled effect estimates and found no single study exerted significant effect on the overall effect estimate of the meta-analysis. Details of the sensitivity analysis are provided in Supplementary Table S2.

## Discussion

This systematic review and meta-analysis summarizes the current knowledge on the association between qualitative and quantitative estimates of microbial agents within the indoor environment and respiratory symptoms among children under the age of 5 years. To the best of our knowledge, our meta-analysis is the most comprehensive overview to investigate whether exposure to indoor microbial aerosols is associated with respiratory health of children under the age of 5 years. First, we observed that exposure to a combination of *Aspergillus*, *Penicillium* and *Cladosporium* species increased the risk of wheeze by 67%. Secondly, the association of indoor microbial exposure with allergic rhinitis was not significant. Thirdly, exposure to microbial agents such as endotoxins was protective against asthma, although the estimate was from two studies.

The combined analysis revealed an increased risk of wheeze when children under 5 were exposed to a combination of *Aspergillus* and *Penicillium* species, which was reduced by the addition of *Cladosporium* species to the model. This suggests that microbial interaction within the indoor environment may play a role in the respiratory health of children under the age of 5 years. These specific fungi within the indoor environment have also been shown to be associated with an increased risk of wheeze [17, 36] and asthma [24, 35, 50] in longitudinal studies. A similar meta-analysis on fungal exposure and respiratory health in children aged 6–12 years [51] compares well to the pooled estimate reported in our meta-analyses. Relevant studies both in vitro and in vivo have demonstrated that repeated activation of immune responses and inflammation from fungal exposures may contribute to inflammation-related diseases, and the resulting inflamed mucosal tissue may provide a diminished barrier to respiratory infections [17]. Also, prolonged exposure to aerosolized fungal components mainly target the respiratory and nervous system causing specific pathological changes in the host characterized by inflammation and continuous activation of immune responses as a result of fungal exposures may contribute to inflammation-related diseases [52]. Our review considered exposure to visible molds only or in association with dampness, but excluded studies considering dampness alone. Indeed, mold and dampness exposures are often connected, leading to increased fungal growth and correlated microbial exposures, such as fungal spores, hyphae, fragments [53], microbial volatile organic compounds [54, 55], mycotoxins [56], house dust mite allergens [57] and endotoxins [58, 59].

The definitions of respiratory outcomes among children < 6 years of age are often poorly described and confusing, thereby making the diagnosis of the disease in preschool children difficult [60]. As a result, the European Respiratory Society task force proposed the use of terms such as “episodic (viral) wheeze” (among children with recurrent wheeze and who are well between episodes) and “multiple-trigger wheeze” (among children who wheeze both during and after discrete episodes) [61]. In fact, some other definitions have also been used to describe the different phenotypes of preschool wheezing disorders such as the presence of transient early wheezing in children < 3 years, non-atopic wheezing in children aged 3–6 years, and IgE-mediated wheeze in older children [62]. More recent studies have suggested that these definitions may reflect disease severity and that they are likely to vary with time [63]. Specific factors responsible for the development of respiratory diseases in children < 6 years has not been identified; however, interactions between the environment and genetic factors of each individual play a vital role [64]. These factors include infections, atopy, prematurity, exposure to tobacco smoke, exposure to elevated levels of air pollution or family history of asthma [62, 65, 66]. A recent meta-analysis reported an increased risk of lower respiratory tract infection among under-five children as a result of increased exposure to indoor microbes with emphasis on detailed microbial characterization using modern molecular techniques [67].

It was interesting to discover that none of the studies included in the present meta-analysis employed molecular-based techniques in the analysis of microbial agents. Although, studies have investigated the burden of indoor microbial exposures using sequencing-based assessment [9, 68–70] but very few demonstrated a link with disease epidemiology such as respiratory outcomes as revealed in the present study. This is probably due to the complexity of the microbial exposure and the lack of clear understanding of the mechanism involved in the association between indoor microbial agents and disease outcomes.

Recent studies have emphasized the protective effect of exposure to endotoxins [25, 26, 71–73] against respiratory allergies and allergic asthma. Our findings corroborate these reports but contradicts some findings among older children [74–76] and adults [77–79]. A previous study reported that exposure to endotoxin has been associated with reduced risk of childhood atopy but an increased risk of wheeze [80]. A comprehensive meta-analysis confirmed this contradiction with respect to endotoxin exposure [73]. Among pre-school children, in whom asthma is more strongly associated with atopy, endotoxin exposure resulted in reduced risk, whereas among infants and toddlers with virus-triggered wheeze, endotoxin increased the risk [73]. Similar findings were reported from the survey carried out by the National Health and Nutrition Examination Survey study enrolling more than 6,000 subjects across the United States. They found that endotoxin was a risk for wheeze but not asthma [59]. Less is known about other bacterial and fungal exposures in indoor urban homes. In the Boston cohort increased levels of muramic acid as a marker for gram-positive bacteria were inversely associated with current asthma but not with allergic sensitization [80]. However, the mechanisms are still not fully understood. Possible explanation indicate that endotoxin is a potent inducer of interleukin-12 and interferon gamma, which downregulate the production of T-lymphocyte helper 2 (Th2) cells involved in the development of allergic diseases [81]. Besides, the potential of T-lymphocyte helper 1 (Th1) inducers like endotoxin and other microbial exposures to mitigate allergy and asthma is consistent with clinical studies. Overall, evidence of the mechanism of association between exposure to indoor microbes and respiratory outcomes is limited. Nevertheless, further longitudinal studies of the effect of early-life exposure to endotoxins on subsequent child health will be needed to understand this mechanism more fully.

In addition, studies included in the meta-analyses have used proxy measures for estimating respiratory health outcome, such as outcome obtained from parental or other caregiver interviews, questionnaires, and medical records. None of the studies quantified indoor microbial contamination to the species level using molecular techniques, which restricted analyses to the fungal genera and potentially underestimate exposures. Also, the differences in the RRs across the included studies could have also contributed to the high heterogeneity. Regardless of these limitations, this study has provided sufficient evidence required in designing future longitudinal studies to further investigate and explain the mechanism involved in the exposure–response relationship between indoor microbial exposure and respiratory symptoms among children under 5.

## Conclusions

Indoor microbial aerosol exposures increase the risk of respiratory symptoms such as wheeze and allergic rhinitis but protect against asthma in children under 5. More research regarding these relationships is required using modern analytical approaches such as molecular-based sequencing techniques to better inform/advise parents, form guidelines to reduce exposure to microbial agents within the indoor environment and provide useful intervention strategies for managing the impact of exposure to microbial agents in association with respiratory symptoms among children under the age of 5 years.

## Supplementary Information

**Additional file 1: Table S1.** Quality Assessment of studies included in the meta-analysis using the Newcastle-Ottawa Scaling. **Table S2.** Leave-one-out sensitivity Analysis of *pooled* effect estimates of studies included in the meta-analysis. **Figure S1.** Funnel plots with pseudo-95% confidence limit for any IM exposure and wheeze among U-5C (A); TFC and wheeze (B); *Aspergillus* spp. and wheeze (C); *Penicillium* spp. and wheeze (D); *Cladosporium* spp and wheeze (E); Endotoxin and wheeze (F). **Figure S2.** Funnel plots with pseudo-95% confidence limit for any IM exposure and allergic rhinitis among U-5C (A); visible mold and allergic rhinitis (B); Endotoxin and allergic rhinitis (C). **Figure S3.** Funnel plots with pseudo-95% confidence limit for any IM exposure/endotoxin and asthma among U-5C.

## Data Availability

All data generated or analysed during this study are included in this published article and its additional files.

## Abbreviations

CI: Confidence interval
NOS: Newcastle-Ottawa Scale
OR: Odds ratio
RR: Relative risk
SE: Standard error
EEs: Summary effect estimates
*P*: *P*-Value
Th1: T-lymphocyte helper 1
Th2: T-lymphocyte helper 2
CD14: Cluster of differentiation 14
PRISMA: Preferred Reporting Item for Systematic Review and Meta-analyses
