# Supplementary material for: Association of indoor microbial aerosols with respiratory symptoms among under-five children: a systematic review and meta-analysis
Source: Environ Health. 2021 Jul 1;20:77. doi: 10.1186/s12940-021-00759-2 (PMC8252236; doi:10.1186/s12940-021-00759-2)
Supplement: Supplementary file 1 — Additional file 1: Table S1. Quality Assessment of studies included in the meta-analysis using the Newcastle-Ottawa Scaling. Table S2. Leave-one-out sensitivity Analysis of pooled effect estimates of studies included in the meta-analysis. Figure S1. Funnel plots with pseudo-95% confidence limit for any IM exposure and wheeze among U-5C (A); TFC and wheeze (B); Aspergillus spp. and wheeze (C); Penicillium spp. and wheeze (D); Cladosporium spp and wheeze (E); Endotoxin and wheeze (F). Figure S2. Funnel plots with pseudo-95% confidence limit for any IM exposure and allergic rhinitis among U-5C (A); visible mold and allergic rhinitis (B); Endotoxin and allergic rhinitis (C). Figure S3. Funnel plots with pseudo-95% confidence limit for any IM exposure/endotoxin and asthma among U-5C. [file 12940_2021_759_MOESM1_ESM.docx]

**SUPPLEMENTARY MATERIALS**

Table S1: Quality Assessment of studies included in the meta-analysis using the Newcastle-Ottawa Scaling

| **Studies** | **Selection** | | | | **Comparability** | | **Outcome/Exposure** | | | **Total Score** | **Score (%)** | **Risk** |
| --- | --- | --- | --- | --- | --- | --- | --- | --- | --- | --- | --- | --- |
|  | **1** | **2** | **3** | **4** | **1** | **2** | **1** | **2** | **3** |  |  |  |
| Gent et al., 2002 | * | * | * | * | * | * | - | * | * | 8 | 89 | ꜜ |
| Rosenbaum et al., 2010 | - | * | * | * | * | * | * | * | * | 8 | 89 | ꜜ |
| Horick et al., 2006 | * | * | * | * | * | * | - | - | * | 7 | 78 | ꜛ |
| Park et al., 2001 | * | * | * | * | * | * | * | * | * | 9 | 100 | ꜜ |
| Harley et al., 2009 | * | * | * | * | * | * | * | * | * | 9 | 100 | ꜜ |
| Litongua et al., 2002 | - | * | * | * | * | * | - | * | * | 7 | 78 | ꜛ |
| Campo et al., 2006 | * | * | * | * | * | * | - | * | * | 8 | 89 | ꜜ |
| Gillespie et al. 2006 | * | * | * | * | * | * | - | * | * | 8 | 89 | ꜜ |
| Perzanowski et al. 2006 | - | * | * | * | * | * | - | * | * | 7 | 78 | ꜛ |
| Karvonen et al. 2012 | * | * | * | * | * | * | - | * | - | 7 | 78 | ꜛ |
| Stark et al., 2005 | * | * | * | * | * | * | * | * | * | 9 | 100 | ꜜ |
| Biagini et al., 2006 | - | * | * | * | * | * | - | * | * | 7 | 78 | ꜛ |
| Bolte et al., 2003 | * | * | * | - | * | * | - | * | * | 7 | 78 | ꜛ |
| Douwes et al., 2006 | * | * | * | - | * | * | * | - | * | 7 | 78 | ꜛ |
| Phipatanakul et al., 2005 | * | * | * | - | * | * | - | * | * | 7 | 78 | ꜛ |
|  |  |  |  |  |  |  |  |  |  |  |  |  |
| **Note:**  Low Risk of Bias = ≥80% = ꜜ  High Risk of Bias = <80% = ꜛ | | | | | | | | | | | | |

| \| **Table S2: Leave-one-out sensitivity Analysis of *pooled* effect estimates of studies included in the meta-analysis** \| \| \| \| \| \| \| --- \| --- \| --- \| --- \| --- \| --- \| \| **Study ID; Year/Country** \| **N** \| ***I*^2^ (%)** \| ***Summary EE* [95%CI]** \| ***P*-value** \| **Weight (%)** \| \| **Wheeze** \| \| \| \| \| \| \| All Studies \| **12** \| **78** \| **1.20 [1.05, 1.38]** \| **0.009** \| **100.0** \| \| Gent et al., 2002/USA \| 11 \| 77 \| 1.18 [1.02, 1.36] \| 0.02 \| 9.6 \| \| Rosenbaum et al., 2010/USA \| 11 \| 78 \| 1.18 [1.03, 1.35] \| 0.02 \| 3.2 \| \| Horick et al., 2006/USA \| 11 \| 79 \| 1.18 [1.03, 1.36] \| 0.02 \| 3.6 \| \| Park et al., 2001/USA \| 11 \| 79 \| 1.20 [1.03, 1.40] \| 0.02 \| 10.1 \| \| Harley et al., 2009/USA \| 11 \| 78 \| 1.18 [1.02, 1.35] \| 0.02 \| 7.2 \| \| Litongua et al., 2002/USA \| 11 \| 79 \| 1.18 [ 1.03. 1.36] \| 0.02 \| 5.9 \| \| Campo et al., 2006/USA \| 11 \| 79 \| 1.24 [ 1.08, 1.42] \| 0.003 \| 5.0 \| \| Gillespie et al. 2006/Europe \| 11 \| 79 \| 1.20 [1.03, 1.40] \| 0.02 \| 10.2 \| \| Perzanowski et al. 2006/USA \| 11 \| 80 \| 1.23 [1.05, 1.43] \| 0.01 \| 10.4 \| \| Karvonen et al. 2012/Europe \| 11 \| 61 \| 1.24 [1.09, 1.41] \| 0.001 \| 11.8 \| \| Bolte et al., 2003/Europe \| 11 \| 79 \| 1.19 [1.03, 1.38] \| 0.02 \| 9.9 \| \| Douwes et al., 2006/Europe \| 11 \| 79 \| 1.24 [1.08, 1.42] \| 0.003 \| 5.3 \| \| Phipatanakul et al., 2005/USA \| 11 \| 78 \| 1.18 [1.02, 1.36] \| 0.02 \| 7.9 \| \| **Allergic rhinitis** \| \| \| \| \| \| \| All Studies \| 3 \| 70 \| 1.18 [0.94, 1.48] \| 0.16 \| 100.0 \| \| Gillespie et al., 2006/Europe \| 2 \| 76 \| 1.34 [0.91, 1.95] \| 0.13 \| 31.8 \| \| Perzanowski et al., 2006/USA \| 2 \| 78 \| 1.32 [0.87, 2.01] \| 0.19 \| 35.8 \| \| Stark et al., 2005/USA \| 2 \| 13 \| 1.03 [0.91, 1.17] \| 0.66 \| 23.1 \| \| Biagini et al., 2006/USA \| 2 \| 76 \| 1.14 [0.90, 1.44] \| 0.29 \| 9.3 \| \| **Asthma** \| \| \| \| \| \| \| All Studies \| 1 \| 58 \| 0.78 [0.62, 0.99] \| 0.04 \| 100.0 \| \| Karvonen et al. 2012/Europe \| 0 \| # \| 0.67 [0.51, 0.89] \| 0.006 \| 62.3 \| \| Douwes et al., 2006/Europe \| 0 \| # \| 0.86 [0.75, 1.00] \| 0.04 \| 37.7 \| |
| --- | --- | --- | --- | --- | --- | --- | --- | --- | --- | --- | --- | --- | --- | --- | --- | --- | --- | --- | --- | --- | --- | --- | --- | --- | --- | --- | --- | --- | --- | --- | --- | --- | --- | --- | --- | --- | --- | --- | --- | --- | --- | --- | --- | --- | --- | --- | --- | --- | --- | --- | --- | --- | --- | --- | --- | --- | --- | --- | --- | --- | --- | --- | --- | --- | --- | --- | --- | --- | --- | --- | --- | --- | --- | --- | --- | --- | --- | --- | --- | --- | --- | --- | --- | --- | --- | --- | --- | --- | --- | --- | --- | --- | --- | --- | --- | --- | --- | --- | --- | --- | --- | --- | --- | --- | --- | --- | --- | --- | --- | --- | --- | --- | --- | --- | --- | --- | --- | --- | --- | --- | --- | --- | --- | --- | --- | --- | --- | --- | --- | --- | --- | --- | --- | --- | --- | --- | --- | --- | --- | --- | --- | --- | --- | --- | --- | --- | --- | --- | --- | --- | --- | --- | --- | --- | --- | --- | --- | --- | --- | --- | --- | --- |

|  |  |  |
| --- | --- | --- |
|  |  |  |
|  |  |  |
|  |  |  |
|  |  |  |

| **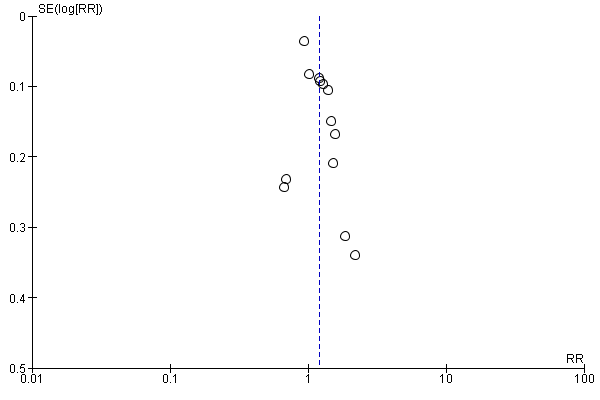** | **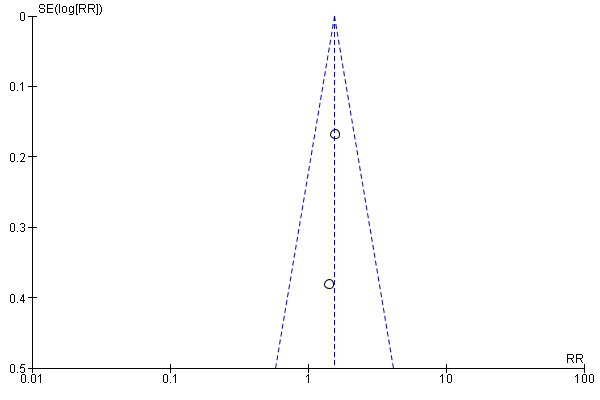** | **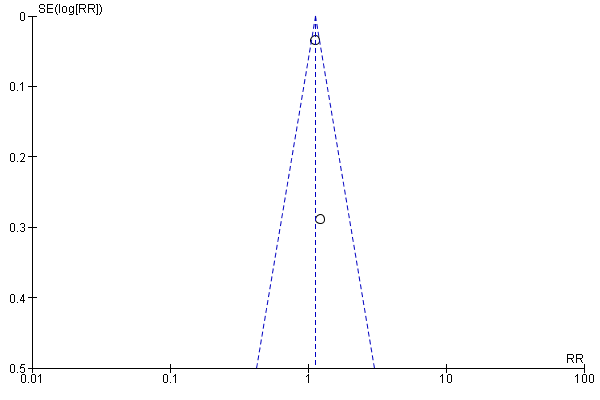** |
| --- | --- | --- |
| **A.** | **B.** | **C.** |
| **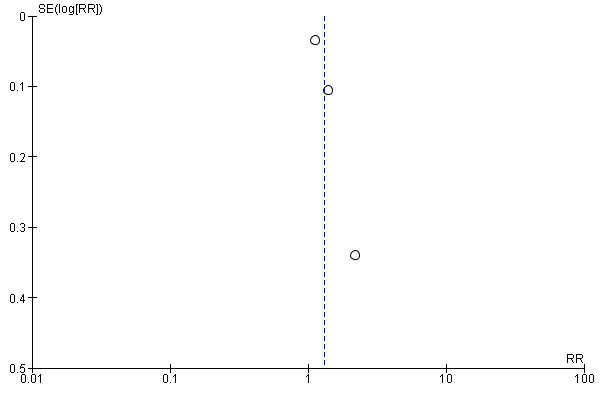** | **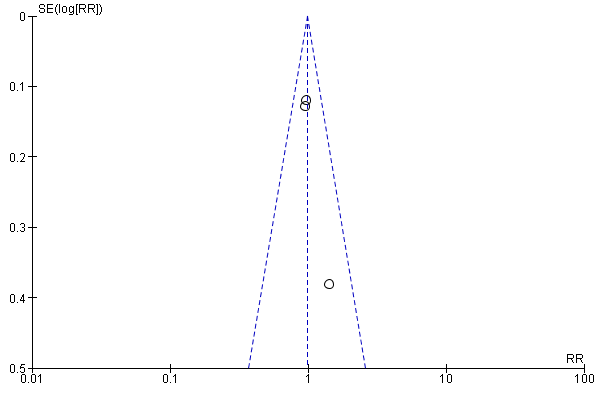** | **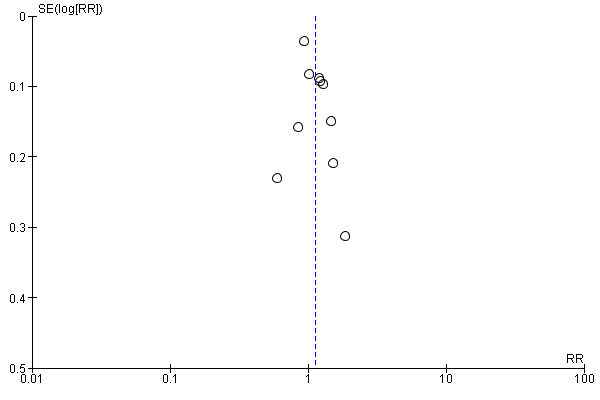** |
| **D.** | **E.** | **F.** |
|  |  |  |
| **Figure S1**: Funnel plots with pseudo-95% confidence limit for any IM exposure and wheeze among U-5C (A); TFC and wheeze (B); *Aspergillus* spp. and wheeze (C); *Penicillium* spp. and wheeze (D); *Cladosporium* spp and wheeze (E); Endotoxin and wheeze (F)   \| **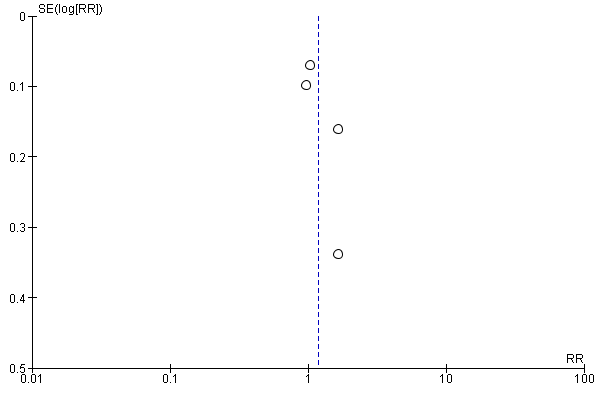** \| **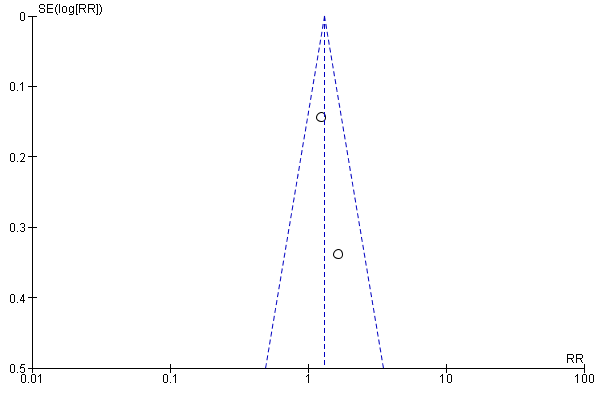** \| **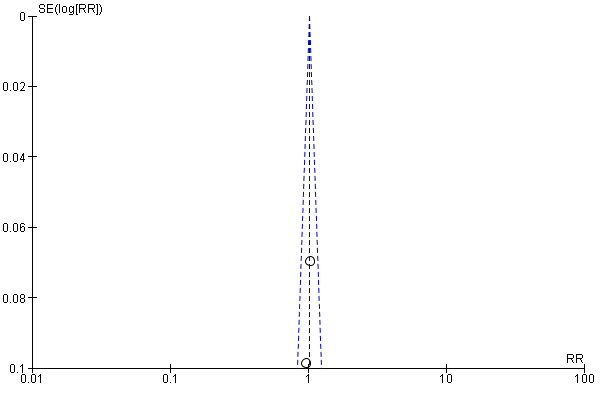** \| \| --- \| --- \| --- \| \| **A.** \| **B.** \| **C.** \| \|  \|  \|  \| \| **Figure S2**: Funnel plots with pseudo-95% confidence limit for any IM exposure and allergic rhinitis among U-5C (A); visible mold and allergic rhinitis (B); Endotoxin and allergic rhinitis (C). \| \| \|   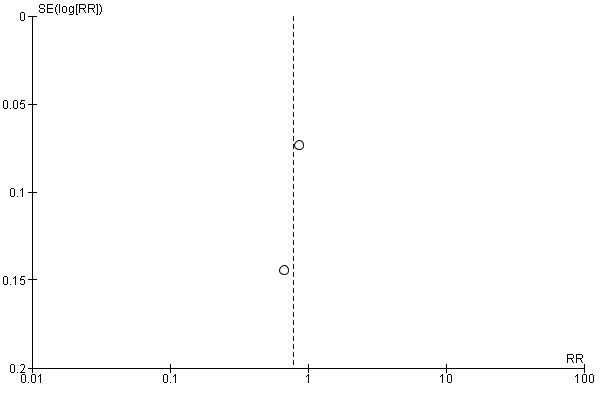  **Figure Figure S3**: Funnel plots with pseudo-95% confidence limit for any IM exposure/endotoxin and asthma among U-5C | | |
